# Tolerance of four grain legume species to waterlogging, hypoxia and anoxia at germination and recovery

**DOI:** 10.1093/aobpla/plab052

**Published:** 2021-08-19

**Authors:** Edi Wiraguna, Al Imran Malik, Timothy David Colmer, William Erskine

**Affiliations:** 1 UWA School of Agriculture and Environment, The University of Western Australia, 35 Stirling Highway, Crawley, WA 6009, Australia; 2 Centre for Plant Genetics and Breeding, The University of Western Australia, 35 Stirling Highway, Crawley, WA 6009, Australia; 3 Institute of Agriculture, The University of Western Australia, 35 Stirling Highway, Crawley, WA 6009, Australia

**Keywords:** Anoxia, germination, hypoxia, legume species, seeds, soil waterlogging

## Abstract

Legume seeds, when relay sown following rice, may suffer from soil waterlogging and the associated hypoxia or even anoxia. This study evaluated the tolerance of grain legume species, grass pea (three genotypes), lentil (two genotypes), faba bean (two genotypes) and field pea (one genotype), to soil waterlogging in a glasshouse, to anoxia and hypoxia in temperature-controlled room at germination and seedling stages. Changes in oxygen in the surface layers of soil, with time after waterlogging, were measured by microelectrode profiling. The soil profiling showed that soil oxygen declined and then stabilized by the fourth day after waterlogging and oxygen was not detected at 8 mm below the soil surface. Germination of seeds under waterlogging for up to 12 days and seedling survival after the soil was drained for up to 36 days were measured in pot experiments. Seed germination and/or survival in anoxia (N_2_-flushed solutions) and hypoxia (1.0 and 2.5 kPa oxygen) were evaluated, and so were post-anoxia or post-hypoxia recoveries, all in comparison with aerated controls. Lentil had higher seedling emergence (55 %) than the other species during soil waterlogging. However, lentil had lower seedling survival (9 %) than grass pea (28 %) during recovery following soil drainage. Grass pea seeds were more tolerant of anoxia and of hypoxia than the seeds of the three other species. In conclusion, grass pea, with higher percent germination and seedling survival during recovery, is more tolerant to waterlogging and subsequent soil drainage than the three other grain legume species. Grass pea was also more tolerant of hypoxia and of anoxia at the seed germination stage. These findings demonstrate the superior waterlogging tolerance of grass pea in relay sowing, as compared with the other grain legumes.

## Teaser

This study evaluated the tolerance of seeds of lentil, faba bean, field pea and grass pea to soil waterlogging, anoxia and hypoxia. The soil profiling of soil waterlogging showed that oxygen declined and then stabilized by the fourth day after waterlogging and oxygen was not detected at 8 mm below the soil surface. Lentil had higher seedling emergence (55 %) than the other species during soil waterlogging. However, lentil had lower seedling survival (9 %) than grass pea (28 %) during recovery following soil drainage. Grass pea seeds were more tolerant of anoxia and hypoxia than the seeds of the three other species.

## Introduction

Grain legume crops, a key dietary protein source for many people, are intolerant to waterlogging (Belford *et al.* 1980; Solaiman *et al.* 2007; Zaman *et al.* 2018). The effect of waterlogging on grain legume production, which can be a reduced yield or even crop failure, depends on the growth stage and duration of the stress (Belford *et al.* 1980; Zaman *et al.* 2018). Grain legumes are often exposed to waterlogging at germination as relay sown crops in rice in the Eastern Gangetic Plains of Bangladesh, Eastern India and Nepal, especially when unseasonal rain occurs (Gupta and Bhowmick 2005; Ali *et al.* 2009; Malik *et al.* 2015).

Relay sowing, a practice of hand broadcasting grain legume seeds into a standing rice crop 2 weeks prior to rice harvest, allows an earlier calendar date for harvest of the grain legume grown as part of the crop sequence (Ali *et al.* 2009; Malik *et al.* 2016). This relay practice avoids tillage before sowing and maximizes access to residual soil water by the grain legume crop. However, in relay sowing systems, legume seeds are often exposed to excess soil water (i.e. waterlogging) and consequently, seeds are subjected to low oxygen (hypoxia) and/or no oxygen (anoxia) that can lead to germination failure (Frenzel *et al.* 1992; Malik *et al.* 2016; Zaman *et al.* 2018).

Tolerance to waterlogging varies between and within grain legume species. Variation within legume species during vegetative stage was identified in faba bean (Solaiman *et al.* 2007). There is a variation amongst legumes species with respect to tolerance to transient waterlogging with the rank as (% of controls): faba bean (*Vicia faba*) > yellow lupin (*Lupinus luteus*) > grass pea (*Lathyrus sativus*) > narrow-leafed lupin (*Lupinus angustifolius*) > chickpea (*Cicer arietinum*) > lentil (*Lens culinaris*) > ﬁeld pea (*Pisum sativum*) (Solaiman *et al.* 2007). During germination stage, variation within legume species was identified in lentil (Wiraguna *et al.* 2017), field pea (Zaman *et al.* 2019) and grass pea (Wiraguna *et al.* 2020). Malik *et al.* (2015) demonstrated variation of waterlogging tolerance following aerobic germination between and within legume species and found that grass pea was more waterlogging-tolerant than lentil and field pea. This study extends the previous study of Malik *et al.* (2015) of waterlogging tolerance of three legume species by including a comparison with faba bean, additional genotypes (from five to eight genotypes) with different seed size, evaluating responses of the seeds to anoxia durations and to different levels of hypoxia, as well as taking soil oxygen profiles in the surface layer where the seeds reside.

There is variation in tolerance of seeds to anoxia between and within species during germination. For examples, faba bean is more anoxia-tolerant than field pea (Crawford 1977), and rice (*Oryza sativa*) is more anoxia-tolerant than wheat (*Triticum aestivum*) (Perata *et al.* 1992; Shingaki-Wells *et al.* 2011). There is limited information on variation within species in tolerance to anoxia at germination, except for rice. Tolerant and intolerant rice genotypes germinated under anoxia, but a tolerant genotype had a faster coleoptile elongation than an intolerant one (Gibbs *et al.* 2000). This is because tolerant genotypes of rice are able to hydrolyse starch in the endosperm as a source of substrate for the production of energy via ethanolic fermentation, which in turn enables the coleoptile to elongate, while intolerant genotypes are not as proficient in starch hydrolysis (Huang *et al.* 2003).

The duration of waterlogging has diverse effects on seed germination depending on the species. For example, waterlogging for 6 and 9 days reduced germination of lentil genotype 70860 by 40 and 80 %, respectively (Wiraguna *et al.* 2017). Waterlogging for 2 days decreased germination of lupin genotype Gungurru by 27 %, and the seeds failed to germinate after 4 days of waterlogging (Sarlistyaningsih *et al.* 1995). Waterlogging for 4 and 8 days reduced germination by 6 and 45 % for field pea genotype Kaspa (Zaman *et al.* 2018).

Crop species also show different responses to hypoxic conditions during germination (Al-Ani *et al.* 1982, 1985; Nakajima *et al.* 2015; Yasin and Andreasen 2016). Seeds of wheat and field pea geminated at a very low oxygen partial pressure of 0.1 kPa but seeds of flax (*Linum usitatissimum*) failed to germinate under the same conditions (Al-Ani *et al.* 1985). In vegetables, the germination rate of cauliflower (*Brassica oleracea botrytis*) was reduced by 40 % after being subjected to hypoxia at 10 kPa oxygen, while the germination rate of broccoli (*Brassica oleracea italica*) was not affected at 10 kPa oxygen (Yasin and Andreasen 2016). However, information of germination responses to hypoxia amongst various grain legume crops (such as the lentil, field pea, grass pea and faba bean studied here) is limited.

The following experiments were designed to test the hypotheses that: (i) there is variation between and within grain legumes (lentil, field pea, grass pea and faba bean) for tolerance of differing durations of soil waterlogging (up to 12 days) at germination, as well as in recovery growth following drainage; (ii) there is variation for tolerance of grain legume seeds to anoxia (up to 9 days) applied at imbibition and also differences in recovery ability following subsequent re-aeration; and (iii) the seeds of the grain legumes differ in tolerance of hypoxia during germination.

## Materials and Methods

This study comprised three experiments. Experiment 1 was to elucidate temporal and spatial changes in oxygen during soil waterlogging in pots in a temperature-controlled room. Experiment 2 was to identify the effects of duration of soil waterlogging—followed by recovery upon drainage, where seeds of four grain legume species (and genotypes within species) could germinate and survive to the seedling stage in pots of soil in a glasshouse. Experiment 3 tested the survival of imbibed seeds during anoxia and hypoxia in controlled laboratory conditions.

A preliminary experiment was conducted to test seed viability of each genotype. Two layers of filter paper (Whatman no. 50) saturated with deionized water (DI water) were put in a Petri dish (90 mm diameter). Ten seeds were placed on top of wet filter papers in each Petri dish and one layer of DI water-saturated filter paper was put on the top of the seeds. Deionized water was sprayed as required (1–2 times a day) to ensure adequate moisture for seed germination. The seeds were kept in a temperature-controlled room (25 °C) in the dark for 6 days. Seed viability was calculated as the percentage of seed germination, when radicals emerged at >5 mm in length. There were three replicate Petri dishes of each genotype. The seeds were collected from the previous growing season and stored in a 4 °C cool room.

### Experiment 1: temporal and spatial changes in oxygen during soil waterlogging in controlled laboratory conditions

Measurement of oxygen profiles in Experiment 1 was taken every 1 mm across air (2 mm above water surface), water (~9 mm above the soil surface) and soil (~8 mm below the soil surface) for 10 days. This experiment consisted of the two factors of waterlogging duration (0–10 days) and spatial locations (air, water and soil up to 8 mm below the soil surface) for soil-filled pots (replicated four times), at 25 °C. Black plastic pots (85 × 85 × 180 mm) were each fitted with a transparent plastic bag and soil (~0.7 kg) from Mukinbudin, Western Australia (30°78′S, 118°31′E). The soil was sieved to <2 mm particle size, contained 0.3 % organic carbon, with pH 8.2 and EC 589 µs cm^−1^ of 1:5 w/v soil:DI water (Kotula *et al.* 2015; Zaman *et al.* 2018).

Deionized water (~150 mL) was added in the plastic pots up to a level of ~9 mm above the soil surface and was topped-up daily to maintain the constant water level. The oxygen partial pressure was measured by a Clark-type oxygen mini-electrode with a guard cathode and a tip diameter of 500 µm (OX-500, Unisense, Aarhus, Denmark) connected to a pico-ampere meter (PA2000, Unisense). Calibration of the oxygen mini-electrode was done in DI water at air equilibrium (257.9 µmol L^−1^ or 20.6 kPa) and in anoxic solution (zero oxygen) of 0.5 mM KOH and 8 g L^−1^ ascorbate, as described by Colmer *et al.* (2013). Deionized water in air equilibrium was prepared by bubbling the DI water with air for ~20 min. The oxygen partial pressures in the pots were measured after ~20 min of soil waterlogging for zero (0) day, and then every ~24 h for Day 1–10.

### Experiment 2: the effects of duration of soil waterlogging at germination in the glasshouse

The experiment comprised of two factors: duration of waterlogging and genotypes in a completely randomized design replicated four times. The first factor was duration of waterlogging (0, 3, 6, 9 and 12 days). The second factor was grain legume genotype of the four species—lentil, grass pea, field pea and faba bean from different countries of origin to identify variation in waterlogging tolerance between and within species (Table 1). The experiment was conducted in a glasshouse during late spring (November to December 2017) at The University of Western Australia (31°59′S, 115°49′E) with average day/night maximum and minimum air temperatures of 29.6 ± 0.1 and 25.4 ± 0.1 °C. As Experiment 1 showed that the oxygen level in waterlogged pots stabilized after 4 days of waterlogging, treatment pots were waterlogged for 4 days prior to sowing in Experiment 2.

**Table 1.** Legume species used in Experiment 2 and 3.1, and a subset used in Experiment 3.2, with country of origin, 100 seed weight and seed viability. Means are followed by standard error (*n* = 3). Differences in 100 seed weight and seed viability between genotypes are shown as different letters (*P* < 0.05).

Species	Genotype	Experiment 3.2	Country of origin	100 seed weight (g)	Seed viability (%)
Lentil	Nugget		Australia	3.1 ± 0.1^e^	70 ± 0.3^b^
	70860	X	Bangladesh	1.4 ± 0.2^e^	73 ± 8.9^b^
Grass pea	Chalus	X	Australia	8.9 ± 0.1^d^	100 ± 0.0^a^
	8605		Bangladesh	10 ± 0.9^d^	100 ± 0.0^a^
	Site 41.4		Greece	8.3 ± 0.2^d^	73 ± 6.7^b^
Field pea	Kaspa	X	Australia	17 ± 0.3^c^	70 ± 9.8^b^
Faba bean	Samira	X	Australia	78 ± 0.8^b^	100 ± 0.0^a^
	Early Coles		Australia	111 ± 1.6^a^	100 ± 0.0^a^

Free draining pots (diameter 17.5 cm and height 17 cm) were filled with the same soil (~3.2 kg per pot) as used in Experiment 1. The water content (w/w) at field capacity (i.e. pot capacity when fully drained) was 20.8 %. To achieve waterlogging, each pot was placed into a bigger sealed pot (diameter 20.5 cm and height 19 cm) so that when waterlogged treatments were imposed the water entered the soil in each pot from the base.

Seeds were surface-sterilized with 1 % commercial bleach (active ingredients NaOCl 40 mg L^−1^) for 1 min, washed 3–5 times with DI water. Fungicide (Tetramethylthiuram disulphide) (3.0 g kg^−1^ seeds) (Zaman *et al.* 2018) and rhizobia inoculum group E/F (1.0 g kg^−1^ seeds) were applied to seeds before sowing 10 seeds per pot. The seeds were pressed slightly into the soil surface so that the upper side of each seed was level with the soil surface. Hilum was buried a few millimetre under the soil and faced aside parallel to the soil surface during sowing. The method of gently pressing seed into the soil follows similar research on waterlogging tolerance at germination on field pea (Zaman *et al.* 2018) and grass pea (Wiraguna *et al.* 2020). In the drained control (0 day of waterlogging), DI water (~20 mL) was sprayed three times daily during early sowing (1–4 days after sowing [DAS]) and once daily at the seedling stage (5–36 DAS) to maintain moisture for seed germination and seedling growth. For the waterlogging treatment (3, 6, 9 and 12 days), the water level in pots was maintained ~9 mm above the soil surface so that the seeds were submerged. During drainage after waterlogging, seeds were allowed to emerge and grow under 90 % shade in the first 14 DAS and without shade in the following days (15–36 DAS). Therefore, long duration of waterlogging had less period of re-aeration drainage under 90 % shade than short duration of waterlogging. After 3 days of drainage following waterlogging, DI water was sprayed once a day to provide adequate moisture for seedling growth during stress recovery. The DI water was added to each pot after the first 3 days of drainage to keep soil moisture at field capacity. The pots were re-randomized weekly to minimize positional effects in the glasshouse.

Pots were under 90 % shade (shade cloth covers across the top and sides) for the first 14 days to mimic relay sowing when seeds were under a canopy of mature rice crops (Gupta and Bhowmick 2005). Fourteen days after sowing, seedlings were exposed to 100 % light (640 µmol m^−2^ s^−1^; the natural sunlight inside the glasshouse) by removing the shade cloth. This illuminance change was implemented to mimic removal of a mature rice crop canopy at harvest, typically a fortnight after relay sowing (Gupta and Bhowmick 2005; Malik *et al.* 2016). The commercial fertilizer Diamond Red consisting of 7.3 % nitrogen, 11 % phosphorus, 28 % potassium, 2.8 % sulphur, 0.21 % iron, 0.1 % manganese, 0.08 % boron, 0.06 % zinc and 0.008 % molybdenum was added to each pot (2.03 g per pot) at 3 weeks after sowing. The seedlings were harvested at 36 DAS.

### Experiment 3: the effects of anoxia and hypoxia on germination and subsequent seedling growth during re-aeration in controlled laboratory conditions

#### Experiment 3.1: different durations of anoxia imposed on grain legume seeds at germination.

There were two factors in the Experiment 3.1: (i) seeds of eight genotypes of four grain legume species (Table 1); and (ii) duration of anoxia for 0, 3, 6 and 9 days. The duration of anoxia was shorter than waterlogging duration in Experiment 2 because most germinated seeds had not survived to seedling stage with 12 days of waterlogging in Experiment 2. A completely randomized design with three replicates was used.

Seeds of the eight grain legume genotypes were surface-sterilized as the Experiment 2. Twelve sets of 20 seeds were prepared per genotype, and nine of these sets (the other three sets were the Day 0 aerated controls) placed in small bags made from cheese-cloth containing a glass marble as a weight to ensure each bag remained submerged in solution (0.5 mM CaSO_4_) within a PVC chamber. Each chamber contained 3 L of the solution and eight small bags—one small bag per genotype—and the chambers were sealed except for a small hole which enabled escape of the high-purity N_2_ which was continuously flushed through the solution within each chamber.

The solution of 0.5 mM CaSO_4_ in DI water was made the day before use and placed in the 25 °C room and bubbled with air for 4 h before transferring to the PVC chambers (3 L) and then bubbled with high-purity N_2_ gas for 4 h, so that it was anoxic when the seeds were placed into the solution. The N_2_ gas flushing continued and the chambers remained closed for the duration of the anoxic phase of the experiment and the chambers ensured all seeds remained in darkness. After anoxia for 0-, 3-, 6- and 9-day treatments, seeds were transferred to re-aeration (recovery) on a floating mesh for 19, 16, 13 and 10 days, respectively. A temperature of 25 °C was maintained and the seeds/seedlings remained in darkness except for a brief period of dim light when inspected daily during experimentation. The experiment was terminated at 19 DAS.

#### Experiment 3.2: effects of hypoxia (1.0 or 2.5 kPa oxygen) on grain legume seeds at germination.

There were two factors in Experiment 3.2: (i) legume species with one genotype for each species (Table 1) and (ii) different oxygen partial pressures at 0, 1.0, 2.5 and 20.6 kPa for 6 days followed by aeration (recovery) on a floating mesh for 8 days. Duration of oxygen treatment was 6 days because only a few seeds had survived after 9 days of anoxia and seedling survival was not affected by <3 days of anoxia in Experiment 3.1.

The solution and condition of a controlled temperature room were as described in Experiment 3.1. The solution was bubbled with air for 4 h before transferring to 250-mL flasks. The solution in each of the flasks was then bubbled with a gas mixture of N_2_ and air (regulated by mass flow controllers) for 1 h to achieve oxygen partial pressures of 0, 1.0, 2.5 and 20.6 kPa in a number of individual flasks. Twenty seeds of one species were placed in each of the flasks (i.e. one flask was a replicate of one species; there were three replicates of each species and oxygen concentration combination) and the gas mixtures continued to bubble in the flasks for 6 days. At 3 days, the solution in each flask was replaced with a solution of the same oxygen partial pressures (i.e. pre-bubbled prior to use) to minimize the possible build-up of bacteria in the solution in the flasks. The flasks were sealed with a rubber bung which had a small needle (1.1 mm in diameter) to provide an outlet for the gas mixture which was continuously flushed through each flask. After 6 days, the seeds/seedlings were transferred to a floating mesh on aerated solution to assess recovery. The experiment was terminated at 14 DAS.

### Germination and survival assessments

Germination of seeds was recorded daily for Experiment 2 and 3. Seeds were identified as germinated when the radicle emerged to reach ~2 mm in length for faba bean (Ellis *et al.* 1987) and 5–6 mm for the other legumes (Kranner *et al.* 2010; Wiraguna *et al.* 2017). The number of germinated seeds in each pot was recorded from 3 DAS in Experiment 2; in Experiment 3.1 the recording started at the end of each anoxia treatment period (i.e. during recovery on a floating mesh) and in Experiment 3.2 the recording began from 1 DAS.

In Experiment 2 at final sampling (36 DAS), seedling survival was recorded; shoots and roots were separated. Tissue samples were dried for 72 h at 59 °C and dry mass was determined. Root mass ratio (RMR) was analysed as the proportion of root dry mass to total seedling dry mass.

In Experiment 3.1, the total number of germinated seeds was counted during full aeration on a floating mesh and percent seedling survival was recorded at final sampling. Shoot length and shoot dry mass were measured at the end of the recovery phase (19 DAS).

In Experiment 3.2, germination was recorded during 6 days of oxygen treatments in the flasks. The number of new germinants and surviving seedlings were counted during 8 days of re-aeration on the floating mesh.

### Statistical analyses

The data were analysed by two-way analysis of variance (ANOVA) to identify differences between species, genotypes, treatments and interactions using R-studio version 1.0.136 and GenStat 20th edition (VSN International, UK). Analysis of variance and mean differences in percentage of germination, seedling emergence and seedling survival were applied after accounting for seed viability; i.e., dividing treatment data by the germination percentage of the seed viability test for Experiment 2 (Table 1). In Experiment 3, ANOVA and mean differences in percentage of germination and seedling survival were applied after dividing the number of germinated seeds and seedling survival to initial number of seeds sown. Repeated measures ANOVA was conducted for Experiment 1 by R-studio based on oxygen partial pressure in waterlogged soil for 10 days at between 1 and 8 mm below the soil surface. Means were compared at *P* = 0.05 level using least-significant difference test.

## Results

### Experiment 1: temporal and spatial changes in oxygen during soil waterlogging in controlled laboratory conditions

**Figure 1.**
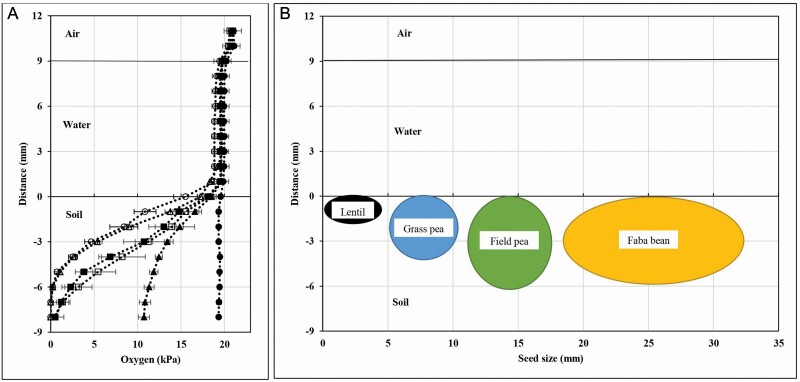
Profiles of oxygen partial pressure in air, water and soil with distance in pots of waterlogged soil in a controlled temperature room (25 °C) at various times for 0 (closed circle, 20 min), 1 day (closed triangle), 2 days (open square), 3 days (closed square), 4 days (open circle) and 10 days (open triangle) (A). Diagram of seed placement of lentil (black), grass pea (blue), field pea (green) and faba bean (yellow), where upper side of the seeds was level with the soil surface at sowing. Sizes of the circles/ovals represent seed sizes of the four grain legume species (B). Means are followed by standard error (*n* = 4) (Experiment 1).

The aim of Experiment 1 was to measure oxygen partial pressure at different depths in waterlogged soil pots. Oxygen partial pressure in air (20.6 kPa) and DI water (19.5 kPa) above the soil surface did not change during 10 days of waterlogging (Fig. 1A). However, oxygen partial pressure was significantly reduced in soil that was waterlogged for 10 days (1–8 mm below the soil surface) (*P* < 0.001). The oxygen partial pressure in waterlogged soil decreased significantly from 19.4 to 2.3 kPa in the first 2 days of waterlogging at a depth of 6 mm below the soil surface (Fig. 1A). The oxygen levels continued to drop until Day 4, at which time the levels stabilized at 4–10 days of waterlogging (Fig. 1A; **see****Supporting Information—Table S1**). After 4 days of waterlogging, the oxygen partial pressure decreased from 10.9 to 0.2 kPa at the depth of 1–6 mm below the soil surface, respectively, and oxygen was not detected at 8 mm below the soil surface.

Seed sizes differed between legume species and lentil had smaller seeds than other species. Small seeds were exposed to higher oxygen levels than the large seeds, because their lower sides were at a shallower soil depth than for the large seeds, whereas the upper side of all seeds would have been exposed to the same oxygen partial pressure (Fig. 1B). For example, small seeds of lentil with a seed diameter of ~2.3 mm were exposed to higher oxygen between 4.6 and 15.5 kPa than large seeds of faba bean with a seed diameter of ~5.9 mm and oxygen partial pressures of 0.2–15.5 at sowing after 4 days of soil waterlogging (Table 2).

**Table 2.** Grain legume species, genotype, seed diameter (seed width; on the minor axis for seeds which were not spherical), seed length (on the major axis for seeds which were not spherical) and oxygen available to the deepest side of seeds at sowing day of eight grain legume genotypes (Experiment 1). Seed thickness was derived from diameter of round (spherical) shapes (field pea and grass pea) and minor axis (seed width) of oval shapes (lentil and faba bean). Means are followed by standard error (*n* = 5). Oxygen experienced at the lower surface of the seed was derived from an approximate value of oxygen partial pressure measured every 1 mm below the soil surface.

Species	Genotype	Seed diameter (mm)	Seed length (mm)	Oxygen experienced at the lower surface of the seeds at sowing (kPa)
Lentil	Nugget	2.6 ± 0.1	5.4 ± 0.1	4.6
	70860	2.1 ± 0.0	3.7 ± 0.1	8.4
Grass pea	Chalus	4.2 ± 0.1	5.6 ± 0.2	2.6
	8605	4.6 ± 0.3	5.9 ± 0.3	0.7
	Site 41.4	3.8 ± 0.2	6.8 ± 0.2	2.6
Field pea	Kaspa	6.2 ± 0.2	6.6 ± 0.2	0.2
Faba bean	Samira	6.4 ± 0.1	17.1 ± 0.5	0.2
	Early Coles	5.6 ± 0.3	20.9 ± 0.7	0.2

### Experiment 2: the effects of duration of soil waterlogging at germination in the glasshouse

The aim of Experiment 2 was to identify different responses in seedling emergence and survival of four legume species (eight genotypes, 1–3 genotypes in each species) to various durations of waterlogging under 90 % shade (i.e. to simulate seeds sown under a rice crop canopy) in the first 14 DAS and no shade from 15 DAS to final sampling (36 DAS).

**Figure 2.**
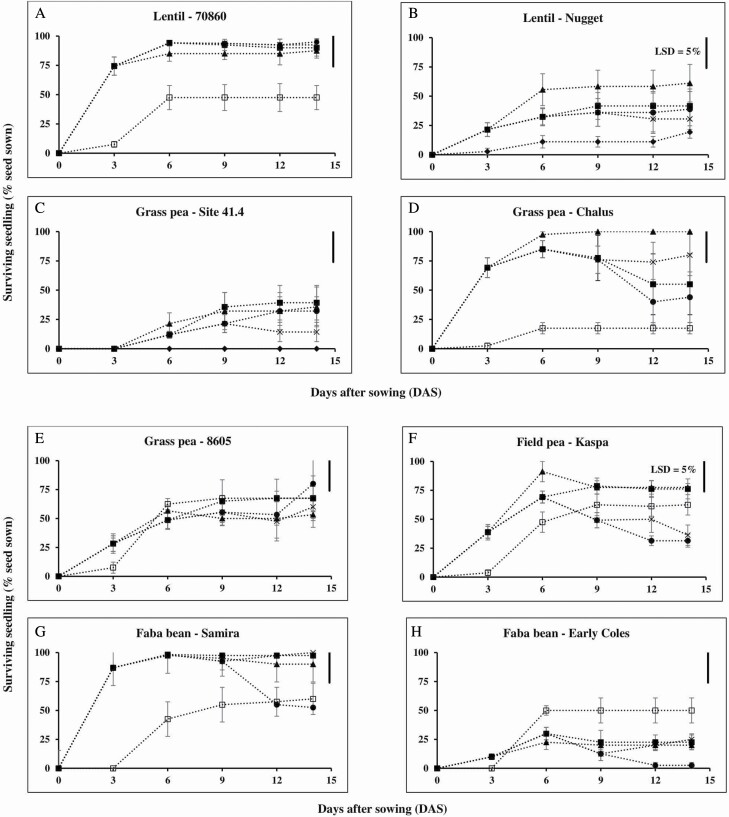
Percentage emergence and survival (corrected for seed viability) of eight grain legume genotypes subject to soil waterlogging for 0 day (open square), 3 days (closed triangle), 6 days (closed square), 9 days (multiplication sign) and 12 days (closed circle) while under 90 % shade for 14 days. Data are means (*n* = 4) of percent surviving seedling ± SE and 95 % confidence interval. The line bars in each graph represent least-significant difference (LSD) at *P* = 0.05 for interaction between the genotype × waterlogging treatment duration with LSD = 26 at 14 DAS. The average day/night temperature was 29.0 ± 0.1/26.8 ± 0.1 °C (Experiment 2).

In the first 14 days (90 % shade), duration of waterlogging had a negative effect on surviving seedling in legume species. There was a lower percent surviving seedling at 0 day of waterlogging (drained pots) than for seeds at 3 days of waterlogging for both lentil genotypes, grass pea genotypes Chalus and Site 41.4, and faba bean genotype Samira (Fig. 2). The lower percentage of seedlings for the drained pots was presumably because water application (i.e. ~60 mL day^−1^) during the first 4 DAS was insufficient for the seeds to fully imbibe and germinate; evaporation of water from the air-exposed upper side of the seeds and from the soil occurred between the water applications to these pots and the seeds dried out.

Legume seeds had different responses to waterlogging duration (and subsequent drainage) in terms of seedling survival during 0, 3, 6, 9 and 12 days of waterlogging followed by 14, 11, 8, 5 and 2 days of recovery upon drainage under 90 % shade, respectively (Fig. 2). Grass pea genotypes showed three different response patterns (tolerant, medium tolerant and intolerant to waterlogging), while lentil and faba bean showed two contrasting responses (tolerant and intolerant to waterlogging).

In lentil, the two genotypes had contrasting responses of seedling survival (Fig. 2A and B). Waterlogging-tolerant lentil genotype 70860 from Bangladesh with small seed size had a similar waterlogging response to grass pea genotype 8605, where seeds germinated under waterlogging and continued to survive during recovery upon drainage (Fig. 2A). In contrast, intolerant lentil genotype Nugget from Australia with large seed size did not germinate during the waterlogging but some seeds then germinated following drainage; overall this genotype showed low percentage of viable seeds that produced living seedlings (55 %) by the end of the first 14 DAS.

In grass pea, all three genotypes showed different responses (response types) to waterlogging followed by drainage with the shade in place for the first 14 DAS (Fig. 2C–E). Type 1, tolerant genotype 8605 germinated under waterlogging and continued to survive during recovery (Fig. 2E). Type 2, genotype Chalus germinated under waterlogging but failed to survive during recovery when exposed to >6 days of waterlogging (Fig. 2D). Type 3, intolerant genotype Site 41.4 had low seedling survival (<39 %) in all waterlogging treatments (Fig. 2C).

In faba bean, seeds of genotype Samira and Early Coles germinated under waterlogging; however, some of the germinated seeds failed to survive during drainage (Fig. 2G and H) similar to that of the medium waterlogging-tolerant grass pea genotype Chalus (Fig. 2D). Genotype Samira was more tolerant to waterlogging (germination 98 %, seedling survival 53 %) than genotype Early Coles (germination 30 % and seedling survival 3 %) after 12 days of waterlogging followed by drainage for 2 days under 90 % shade.

In field pea, seeds of genotype Kaspa germinated under waterlogging and continued to survive during recovery, for 3 and 6 days of waterlogging (Fig. 2F). However, percent seedling survival dropped to 36 and 31 %, when seeds were waterlogged for 9 and 12 days, respectively. This waterlogging response of field pea was similar to grass pea genotype Chalus and faba bean genotype Samira.

**Figure 3.**
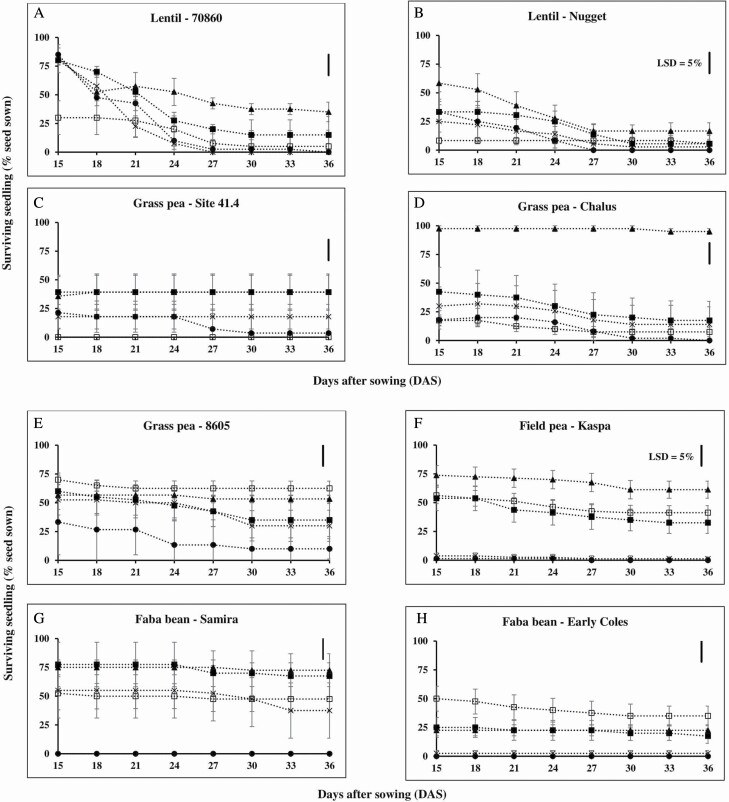
Seedling survival (percentage seeds sown corrected for seed viability) of eight grain legume genotypes during drainage and with shading also removed, following the prior treatments of various waterlogging durations 0 day (open square), 3 days (closed triangle), 6 days (closed square), 9 days (multiplication sign) and 12 days (closed circle). These data from 15 to 36 DAS follow on from the data in Fig. 2. No further germination occurred during this phase of the experiment. The removal of shading was to simulate the situation for relay sown seedlings which are shaded by a rice crop canopy and then exposed to direct sunlight when the rice is harvested. Data are means (*n* = 4) of percent surviving seedling ± SE and 95 % confidence interval. The line bars in each graph represent least-significant difference (LSD) at *P* = 0.05 for interaction between the genotype × waterlogging treatment duration with LSD = 18 at 36 DAS. The average day/night temperature was 29.4 ± 0.1/25.2 ± 0.1 °C (Experiment 2).

In the recovery period after shade removal (15–36 DAS) there was no significant difference in the subsequent seedling survival in the pots with the prior treatment of 3 days of waterlogging, for grass pea (three genotypes), field pea (one genotype) and faba bean (two genotypes); in contrast, both lentil genotypes had significantly reduced seedling survival (Fig. 3A-H). As examples, grass pea genotype Chalus had 98 % seedling survival at the first day of shading removal (15 DAS) and the seedlings survived (95 %) to final sampling (36 DAS) (Fig. 3D), whereas for lentil genotype 70860 the seedling survival dropped from 83 % (15 DAS) to 35 % (36 DAS) (Fig. 3A), for pots which were waterlogged for the first 3 DAS. Medium duration of waterlogging (6 days) gave maximum differentiation among genotypes within species in waterlogging tolerance. In faba bean, for example, seedling survival of genotype Samira only reduced to 68 % but seedling survival of genotype Early Coles dropped to 18 % at 36 DAS after seeds were in waterlogged soil for the first 6 DAS. A long duration of waterlogging (9 and 12 days) reduced seedling survival in all legume genotypes to <40 % at final sampling after the initial waterlogging, drainage and the shade removal (36 DAS).

**Figure 4.**
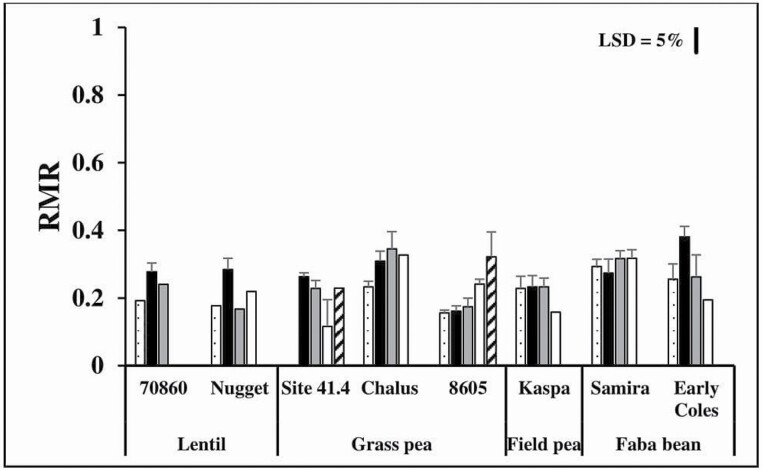
Ratio of dry mass allocation to roots (RMR) of grain legume crops measured at final sampling (36 DAS). The seeds were sown under waterlogged conditions for 0 (dot bar), 3 (black closed bar), 6 (grey closed bar), 9 (open bar) and 12 (striped bar) days, followed by drainage (in 90 % shade until 14 DAS, then without shade on 15 DAS until 36 DAS). The line bar represents least-significant difference (LSD) at *P* = 0.05 for the genotype × treatment interaction with LSD = 0.074. Data are means of seedling survivals ± SE (*n* = 2–4) and 95 % confidence interval with average day/night temperatures of 26.2 ± 0.4/21.4 ± 0.3 °C (Experiment 2).

At the end of the experiment (36 DAS), there was a significant interaction between genotype and waterlogging treatment for RMR (*P* < 0.05) (Fig. 4). The RMR in grass pea genotype 8605 increased from 0.16 to 0.24; whereas the ratio in field pea genotype Kaspa decreased from 0.23 to 0.16 between 0 and 9 days of waterlogging (Fig. 4). In 12 days of waterlogging, only grass pea genotypes 8605 and Site 41.4 survived, and the RMR was higher in 8605 (0.32) than Site 41.4 (0.23). In faba bean, the RMR of genotype Samira was less affected by duration of waterlogging than of genotype Early Coles because genotype Samira had higher RMR at 0.32 than genotype Early Coles at 0.19 after the longest duration of waterlogging (12 days). In lentil, RMR was higher after 3 days of waterlogging than other waterlogging treatments and genotype 70860 had a higher RMR than genotype Nugget (Fig. 4).

### Experiment 3: the effects of anoxia and hypoxia on germination and subsequent seedling growth during re-aeration in controlled laboratory conditions

The aim of Experiment 3.1 was to identify the responses in percent germination and seedling survival of four legume species (1–3 genotypes in each species) to various durations of anoxia (0, 3, 6 and 9 days). The aim of Experiment 3.2 was to assess tolerance of the four legume species (one genotype in each species) to different oxygen partial pressures (0, 1.0, 2.5 and 20.6 kPa). Both experiments were carried out in a temperature-controlled room (25 °C) in the dark.

#### Experiment 3.1: different durations of anoxia imposed on grain legume seeds at germination.

None of the seeds germinated during anoxia, but the duration of anoxia influenced germination and seedling survival during the re-aeration recovery period. There was a significant interaction of germination and seedling survival between species and treatments (*P* < 0.001) but the interaction between genotypes and treatments was not significant. Therefore, species means were used in the analysis of Experiment 3.1.

Grass pea seeds had significantly higher percent germination and seedling survival followed by field pea, faba bean and lentil (*P* < 0.001) during the re-aeration period which followed the anoxia treatments. A long duration of anoxia had a less dramatic impact on germination and seedling survival of grass pea than of the other legume species (Table 3). For example, following 9 days of anoxia, when re-aerated only 16 % of field pea and 19 % of faba bean germinated, but <8 % of field pea and 6 % of faba bean survived as seedlings (% are of the initial number of seeds) by the 10 days of re-aeration at which time the experiment was terminated. In contrast, 25 % of grass pea seeds germinated, and almost all the seedlings survived and grew until the end of experiment (24 % of the initial number of seeds) at 19 DAS (Table 3). Therefore, grass pea was more anoxia-tolerant than the other legume species.

**Table 3.** Means over of genotypes for percentage of germination and seedling survival of lentil (two genotypes), grass pea (three genotypes), field pea (one genotype) and faba bean (two genotypes) seeds in four anoxia durations (0, 3, 6 and 9 days), followed by re-aeration for 19, 16, 13 and 10 days in 0.5 mM CaSO_4_ solution in a dark controlled temperature room (25 °C). The % values, both for germination and seedling survival, are of the initial number of seeds sown (Experiment 3.1). Differences between legume species when exposed to various durations of anoxia were significant (*P* < 0.05) for both traits, and are shown as different letters (least-significant difference, *P* = 0.05) with ± SE (*n* = 3).

Species	Anoxia duration (days)	Germination (%)	Seedling survival (%)
Lentil	0	81 ± 6^ab^	78 ± 6^a^
	3	77 ± 7^ab^	63 ± 9^ab^
	6	9 ± 4^d^	7 ± 3^c^
	9	2 ± 1^d^	0^c^
Grass pea	0	93 ± 3^ab^	89 ± 4^a^
	3	96 ± 2^a^	90 ± 4^a^
	6	81 ± 7^ab^	70 ± 9^a^
	9	25 ± 10^cd^	24 ± 10^bc^
Field pea	0	95 ± 3^ab^	93 ± 4^a^
	3	72 ± 5^abc^	67 ± 2^ab^
	6	58 ± 13^abcd^	33 ± 9^abc^
	9	16 ± 5^cd^	7 ± 4^bc^
Faba bean	0	92 ± 5^ab^	73 ± 11^a^
	3	43 ± 19^bcd^	39 ± 19^abc^
	6	40 ± 14^bcd^	19 ± 4^bc^
	9	19 ± 13^cd^	6 ± 5^c^

**Figure 5.**
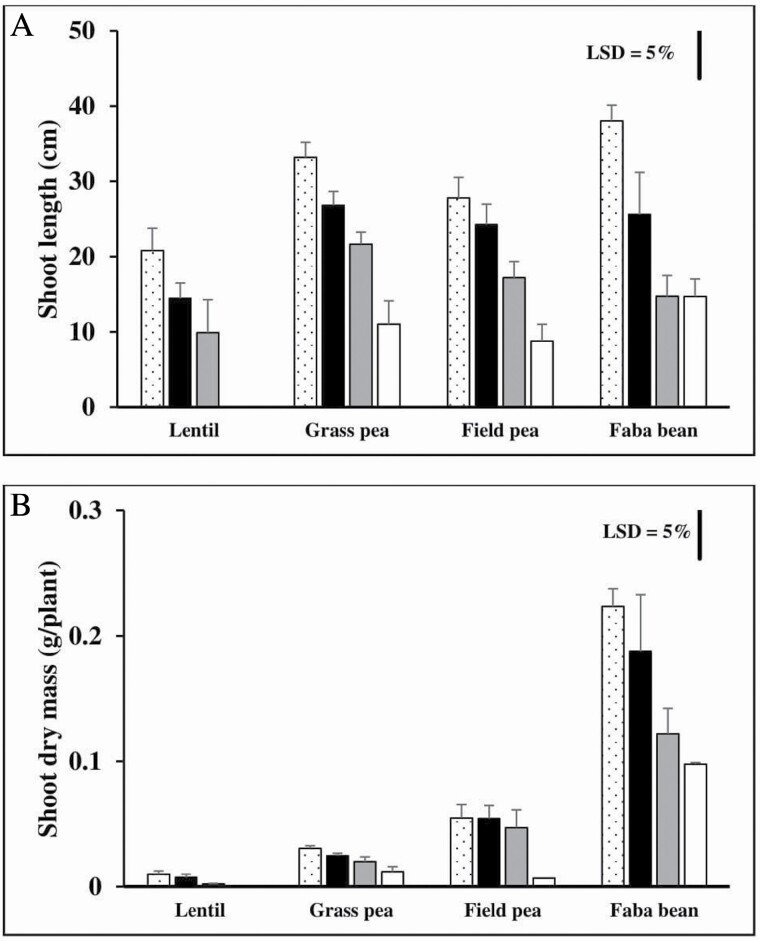
Mean over genotypes for each species of shoot length (A) and dry mass of shoots (B) of lentil (two genotypes), grass pea (three genotypes), field pea (one genotype) and faba bean (two genotypes) in 0.5 mM CaSO_4_ solution with 0 (dot bar), 3 (black closed bar), 6 (grey closed bar) and 9 (open bar) days of anoxia and then re-aeration until 19 DAS, all in darkness. Bar represents least-significant difference (LSD) at *P* = 0.05 for the species × treatment interaction with LSD = 4.47 and 0.04 for shoot length and dry mass, respectively. Data are means (*n* = 2–3) ± SE and 95 % confidence interval. The experiment was in a dark, temperature-controlled room (25 °C) (Experiment 3.1).

Delay in germination until re-aeration impacted on duration of growth. Consequently, seedlings had lower shoot length and dry mass for the longer anoxia treatments (Fig. 5). Therefore, the 0 day of anoxia (control) had the biggest shoot dry mass and longer shoot, whereas seedlings which grew from 9 days anoxia-treated seeds showed the least for both measurements. The shoots were spindly in appearance as the growth had been in darkness.

The shoot length of lentil and faba bean was more affected by the prior anoxia than the shoot length of grass pea and field pea (Fig. 5A). Shoot growth dropped by >30 % relative to control in lentil and faba bean, but the reduction was only <20 % relative to control in grass pea and field pea after 3 days of anoxia and the 16 days re-aeration. The shoot growth reduced further by >50 % relative to control in lentil and faba bean and by 33–39 % relative to control in grass pea and field after seeds being exposed to 6 days of anoxia followed by re-aeration for 13 days. The shoot growth reduction continued to be greater in lentil and faba bean than in grass pea and field pea for a longer duration of anoxia followed by subsequent aeration.

Large seeds showed larger shoot dry mass and longer shoot length than small seeds (Table 1; Fig. 5). For example, large seeded faba bean (~93.5 g in 100 seed weight) had larger (122 mg) and longer shoots (14.7 cm) than small seeded lentil (~2.2 g in 100 seed weight) at 2.2 mg and 9.9 cm, respectively, after 6 days of anoxia and the 13 days of re-aeration (all in darkness).

#### Experiment 3.2: effects of hypoxia (1.0 or 2.5 kPa oxygen) on grain legume seeds at germination.

**Figure 6.**
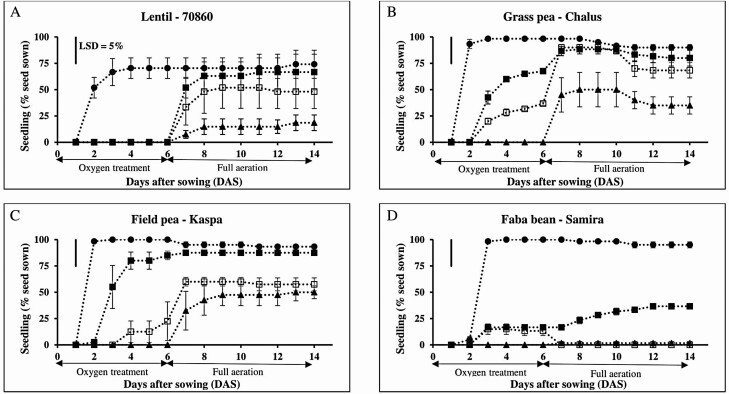
Seedlings (germination and survival) of the percentage of seeds sown (corrected for seed viability) of four grain legume species (one genotype for each species) when subjected to various oxygen treatments at 0 (closed triangle), 1.0 (open square), 2.5 (closed square) and 20.6 (closed circle) kPa for 6 days and followed by full aeration on a floating mesh for 8 days in 0.5 mM CaSO_4_ solution. Data are means (*n* = 3; with 20 seeds per replicate) of percent surviving seedling ± SE and 95 % confidence interval. The line bars in each graph represent least-significant difference (LSD) at *P* = 0.05 for the species × oxygen treatment interaction with LSD = 25 at 14 DAS. The experiment was in a dark, temperature-controlled room (25 °C) (Experiment 3.2).

There was a significant interaction between legume species and oxygen treatments (*P* < 0.05). During 6 days of oxygen treatments imposed when the seeds were first imbibed, seeds of the four legume species did not germinate under anoxia (0 kPa oxygen) but did germinate in the aerated solution with 20.6 kPa oxygen. In the hypoxia treatments (1.0 and 2.5 kPa oxygen) seeds of lentil did not germinate but the other three legume species germinated in both of these oxygen treatments (Fig. 6). Post-treatment recovery was assessed and during full aeration on a floating mesh (7–14 DAS), percent surviving seedling in grass pea was higher than in the other three grain legume species for the seeds previously at an oxygen treatment of 1.0 kPa (Table 4; Fig. 6). For seeds treated with 2.5 kPa oxygen, percent surviving seedling upon return to full aeration on a floating mesh in lentil, field pea and grass pea reduced only by <13 % relative to full aerated controls, while percent surviving seedling in faba bean dropped by 63 % relative to its full aerated control. At the end of the full aeration recovery test (14 DAS), the four legume species had the highest seedling survival after being exposed to 20.6 kPa oxygen (>74 %) but had the lowest percent seedling survival after being treated to anoxia (<50 %) (Table 4).

**Table 4.** Percentage of seedling survival of lentil (70860), grass pea (Chalus), field pea (Kaspa) and faba bean (Samira) seeds in four oxygen treatments (0, 1.0, 2.5 and 20.6 kPa) for 6 days followed by re-aeration on a floating mesh for 8 days in 0.5 mM CaSO_4_ solution in the dark at 25 °C. The % values for seedling survival is of the initial number of seeds sown (Experiment 3.2). Interaction between legume species and oxygen treatments was significant (*P* < 0.05). The different letters indicate which means are significantly different (least-significant difference, *P* = 0.05). Means with ± SE (*n* = 3) are given.

Species	Oxygen partial pressure (kPa)	Seedling survival (%) (total after re-aeration)
Lentil	0	19 ± 7^fg^
	1.0	52 ± 17^de^
	2.5	67 ± 10^bcd^
	20.6	74 ± 10^abcd^
Grass pea	0	50 ± 8^de^
	1.0	90 ± 3^ab^
	2.5	88 ± 4^abc^
	20.6	98 ± 2^a^
Field pea	0	50 ± 8^de^
	1.0	60 ± 4^cde^
	2.5	88 ± 2^abc^
	20.6	100 ± 0^a^
Faba bean	0	2 ± 1^g^
	1.0	15 ± 5^fg^
	2.5	37 ± 2^ef^
	20.6	100 ± 0^a^

In lentil genotype 70860, seeds did not germinate at 0–2.5 kPa oxygen (Fig. 6A). After 1 day of full re-aeration on a floating mesh, the seeds that were previously in low oxygen treatments (0–2.5 kPa) started to germinate. At final sampling (14 DAS), percent surviving seedling increased to 19 % for seeds previously at 0 kPa oxygen, to 48 % for those previously at 1.0 kPa oxygen and to 67 % for those previously at 2.5 kPa oxygen. The controls which received 20.6 kPa oxygen had 74 % germination and all germinated seeds produced seedlings which all survived.

In grass pea genotype Chalus, seeds germinated during 6 days in 1.0–20.6 kPa oxygen but did not germinate under 0 kPa oxygen (Fig. 6B). After 1 day of full re-aeration on a floating mesh (7 DAS), percent germination increased by 45 % for seeds previously at 0 kPa oxygen, by 53 % for seeds previously at 1.0 kPa oxygen and by 19 % for seeds previously at 2.5 kPa oxygen. However, after 8 days of full aeration on a floating mesh (14 DAS), some germinated seeds failed to survive and surviving seedling numbers reduced by 10 % for seeds previously at 0 kPa oxygen, by 22 % for seeds previously at 1.0 kPa oxygen and by 8 % seeds previously at 2.5 kPa oxygen. The controls which received 20.6 kPa oxygen had 98 % germination and surviving seedlings remained high at 90 % at final sampling.

In field pea genotype Kaspa, seeds did not germinate at 0 kPa oxygen but up to 20 % germinated while in 1.0 kPa oxygen and up to 80 % germinated in 2.5 kPa oxygen. Germination in 20.6 kPa oxygen was almost 100 % (Fig. 6C). After 1 day of aeration (7 DAS), germination started and reached 32 % for seeds previously at 0 kPa oxygen, increased by 40 % for seeds previously at 1.0 kPa oxygen and rose by 3 % for seeds previously at 2.5 kPa oxygen (germination was already relatively high at 2.5 kPa oxygen). At final sampling (14 DAS), most germinated seeds had survived, and seedling survival was 50, 58 and 88 % for seeds that were previously at 0, 1.0 and 2.5 kPa oxygen, respectively. The controls which received 20.6 kPa oxygen showed the highest seedling survival at 93 %.

In faba bean genotype Samira, seeds did not germinate during 6 days in 0 kPa oxygen, but some seeds germinated during 6 days in 1.0 and 2.5 kPa oxygen (Fig. 6D). Germination of genotype Samira was greater during 6 days in 20.6 kPa oxygen than in 0, 1.0 and 2.5 kPa oxygen (Fig. 6D). After 1 day of full re-aeration on a floating mesh (7 DAS), seeds previously at 0 kPa oxygen had a low germination percentage of 2 % and seeds previously at 1.0 kPa oxygen failed to germinate, while seeds previously at 2.5 kPa oxygen increased germination percentage but only to 23 %. At final sampling (14 DAS), percent seedling survival was higher for seeds previously at 2.5 kPa oxygen than for seeds previously at 0 and 1.0 kPa. The controls which received 20.6 kPa oxygen showed the highest germination (100 %) and seedling survival (93 %).

## Discussion

Although soil waterlogging, anoxia and hypoxia limit germination in grain legume species, there is variation between and/or within species in tolerance to waterlogging (Malik *et al.* 2015; Wiraguna *et al.* 2017; Zaman *et al.* 2018), anoxia (Crawford 1977) and hypoxia (Al-Ani *et al.* 1985; Yasin and Andreasen 2016). Variation for waterlogging tolerance within legume species was found (Wiraguna *et al.* 2017; Zaman *et al.* 2019); as examples, lentil genotype 70860 and grass pea genotype 8605 were more waterlogging-tolerant than the other lentil (Nugget) and grass pea (Site 41.4 and Chalus) genotypes, respectively (Fig. 2). There was significant variation between the legume species for anoxia and hypoxia tolerances (Table 3; Fig. 6). When legume seeds were exposed to anoxia (Experiment 3.1) or to hypoxia (Experiment 3.2) and then re-aerated, grass pea had higher percent germination and seedling survival than the other three legume species (Tables 3 and 4; Fig. 6).

Oxygen can be a key factor influencing germination of seeds (Vlamis and Davis 1943; Heichel and Day 1972; Al-Ani *et al.* 1982, 1985). A study on *Raphanus sativus* found that seeds failed to geminate when oxygen was reduced to 2 kPa for 6 days (Heichel and Day 1972), while a study of barley (*Hordeum vulgare*) found that germination was reduced to <2 % when seeds were exposed to very low levels of oxygen (0.2 kPa) (Vlamis and Davis 1943). In this study, the four grain legume seeds failed to germinate during anoxia, but some seeds started to germinate after being returned to aerated conditions (Fig. 6). This finding agrees with previous experiments where legume seeds (field pea and faba bean) did not germinate under anoxia and started to germinate only when re-aerated during recovery (Crawford 1977; Rowland and Gusta 1977). During re-aeration post-anoxia, there was significant variation of percent germination and seedling survival between species; and grass pea was more tolerant than the other three species (Table 3). Similarly, there was variation in tolerance of hypoxia. Lentil genotype 70860 did not germinate during 6 days of hypoxia at 1.0 and 2.5 kPa oxygen, while the other legume species germinated (Fig. 6). The seeds of lentil genotype 70860 did not germinate under hypoxia but started to germinate during re-aeration which was probably caused by a barrier located under the seed coat that reduced and/or blocked oxygen diffusion to the lentil seed embryo under hypoxia, similar to that shown for soybean (*Glycine max*) (Tian *et al.* 2005; Sato *et al.* 2019).

Relay sowing can result in germination failure (Zaman *et al.* 2018), which can be caused by a low oxygen concentration in waterlogged soil (Fig. 1; Frenzel *et al.* 1992). In the present study, when seeds were sown to a depth that ensured that the upper surface of the seeds was level with the surface of waterlogged soil, small seeds of lentil genotype 7860 had higher seedling emergence (94 %) than large seeds of faba bean genotype Early Coles (30 %) during 6 days of waterlogging (Figs 1 and 2). The difference in seedling emergence between these legumes was probably because the small seeds of lentil genotype 7860 were exposed to higher oxygen partial pressure at 8.4–15.5 kPa than the large seeds of faba bean genotype Early Coles at 0.2–15.5 kPa (Fig. 1; Table 2). However, when the seeds of lentil and faba bean were exposed to low oxygen partial pressures at 1.0 and 2.5 kPa oxygen for 6 days, the seeds of both legume species had low percent germination at <17 % (Fig. 6), similar to that shown for soybean (Tian *et al.* 2005; Nakajima *et al.* 2015). Therefore, anoxia and hypoxia experiments give an indication that oxygen availability to seeds plays a key role for waterlogging tolerance in small seeds over large seeds when partially buried in the first several millimetres of the soil.

The response of seeds to different oxygen partial pressures varies within species (Tian *et al.* 2005; Nakajima *et al.* 2015). In this study, lentil genotype Nugget with seed diameter of 2.6 mm was exposed to lower oxygen at 4.6–15.5 kPa and had lower percent seedling emergence (i.e. 36 % at 9 days of waterlogging) than lentil genotype 70860 (i.e. 94 % at 9 days of waterlogging) with seed diameter of 2.1 mm and oxygen at 8.4–15.5 kPa (Fig. 1; Table 2). This agrees with germination data for soybean where germination was higher at 15 kPa than at 6 kPa oxygen partial pressure (Al-Ani *et al.* 1985). Heichel and Day (1972) and Vartapetian *et al.* (1983) added that low to absent oxygen partial pressure leads to degradation of mitochondria and tissues of the embryo of corn (*Zea mays*) and results in germination failure and/or suboptimal seedling growth.

Small seeds have higher percent germination than large seeds after waterlogging at germination (Sayama *et al.* 2009; Zaman *et al.* 2018; Wiraguna *et al.* 2020). This experiment also found a similar result compared to previous studies by Zaman *et al.* (2018) and Wiraguna *et al.* (2020), where large seeds of lentil had <40 % seedling emergence while small seeds of lentil showed >85 % seedling emergence after 9 days of waterlogging (Fig. 2). This higher seedling emergence of small seeds over large seeds is probably caused by small seeds being exposed to higher oxygen partial pressure than large seeds since oxygen in waterlogged soil decreases as an increase of soil depth as shown in Fig. 1 and by Frenzel *et al.* (1992). Moreover, large seeds have less oxygen internally owing to a greater diffusion path-length for oxygen to reach all tissues within the seeds (Rolletschek *et al.* 2009). Therefore, small seeds are more tolerant to waterlogging than large seeds.

Variation in root growth during drainage recovery after waterlogging can be used to identify waterlogging tolerance (Laan *et al.* 1989; Malik *et al.* 2015). In the present study, grass pea genotype 8605 (waterlogging-tolerant genotype) formed a larger root system (32 % relative to total biomass) than grass pea genotype Site 41.4 (waterlogging-sensitive genotype; 23 % relative to total biomass) after 12 days of waterlogging and 24 days of recovery (Fig. 4). The root growth after waterlogging at germination can be a crucial factor for the legume species because grass pea, lentil and field pea are often exposed to dry conditions during vegetative and generative stages (Polignano *et al.* 2009; Minta *et al.* 2014; Malik *et al.* 2016; Zaman *et al.* 2018). Moreover, grass pea, lentil and field pea with a long root can absorb more nutrients (Malik *et al.* 2015) and, probably, have a higher yield than grass pea, lentil and field pea with a short root, similar to that shown for soybean (He *et al.* 2017). Therefore, root growth during drainage following transient waterlogging can be identified as a waterlogging tolerance mechanism in grain legume species to survive after waterlogging at germination.

Low oxygen partial pressure (hypoxia) at the soil surface might impact on germination failure of legume seeds (Frenzel *et al.* 1992; Malik *et al.* 2016; Zaman *et al.* 2018). For example, oxygen at the soil surface (0–9 mm) in a rice crop was <5.5 kPa (Frenzel *et al.* 1992) and <33 % of field pea seeds germinated (i.e. relative to control) under relay sowing of mature rice crop (Zaman *et al.* 2018). However, grass pea is possibly tolerant to a low oxygen partial pressure at the soil surface because grass pea seeds had higher percent seedling survival (90 %) than other grain legume species (<60 %) after 6 days of hypoxia at 1.0 kPa followed by 8 days of re-aeration on a floating mesh (Fig. 1; Table 4). Hypoxia tolerance of grass pea genotype Chalus over other grain legume species (lentil genotype 70860, field pea genotype Kaspa and faba bean genotype Samira) is also consistent with grass pea’s reputation for waterlogging tolerance (Gupta and Bhowmick 2005; Malik *et al.* 2015).

Grain legume species are a key dietary protein resource for many people but are intolerant to waterlogging (Solaiman *et al.* 2007; Wiraguna *et al.* 2017; Zaman *et al.* 2018). The incidence of waterlogging is predicted to increase due to an increase in rainfall intensity in some areas, associated with climate change (Sivakumar and Stefanski 2010; Malik *et al.* 2016). Variability among legume species to tolerate waterlogging, anoxia and hypoxia during germination is therefore of importance. We suggest that this variation in waterlogging tolerance, together with the expected increase in this adverse soil condition, should motivate for increased systematic selection of waterlogging, anoxia and hypoxia tolerance in grain legume species.

## Conclusions

There is significant variation between and within grain legume species in tolerance to waterlogging; and between species in anoxia (absence of oxygen) and hypoxia (1.0 and 2.5 kPa oxygen) tolerance during germination, which was also reflected during post-anoxia and -hypoxia re-aeration. Oxygen in soil reduced as the duration of waterlogging increased and oxygen was then only present in the upper 8 mm of a waterlogged soil. Smaller seeds had higher percent seedling emergence than larger seeds, possibly because small seeds were exposed to higher oxygen partial pressure than at least some parts of the larger seeds when sown onto the waterlogging soil. However, the smaller seeds of lentil did not survive during recovery (following drainage) and grass pea with larger seeds had higher seedling survival than lentil during recovery. Seeds did not germinate under anoxia and grass pea seeds showed higher percent germination and seedling survival than the other three legume species during re-aeration to test recovery. Moreover, grass pea had higher percent germination than the other three legume species after being subjected to hypoxia at 1.0 kPa oxygen and similar percent germination to field pea after being subjected to hypoxia at 2.5 kPa oxygen. Therefore, it appears that grass pea is more tolerant to waterlogging, and to anoxia and severe hypoxia (1.0 kPa), than the three other grain legume species and this study clarifies grass pea’s reputation for waterlogging tolerance in relay sowing.

## Supporting Information

The following additional information is available in the online version of this article—

**Table S1.** Multiple comparison based on oxygen partial pressure in waterlogged soil (1–8 mm below soil surface) for 10 days by repeated measures ANOVA.

plab052_suppl_Supplementary_Materials

## Data Availability

No data set was generated in this study.

## Notes

**Agroecology & Environment. Chief Editor:** Amélie Gaudin
