# Supplementary material for: Tolerance of four grain legume species to waterlogging, hypoxia and anoxia at germination and recovery
Source: AoB Plants. 2021 Aug 19;13(4):plab052. doi: 10.1093/aobpla/plab052 (PMC8405847; doi:10.1093/aobpla/plab052)
Supplement: plab052_suppl_Supplementary_Materials [file plab052_suppl_supplementary_materials.pdf]

Supporting Information

Table S1. Multiple comparison based on oxygen partial pressure in waterlogged soil (1-8 mm below soil surface) for 10 days by repeated measure ANOVA

Duration of waterlogging (days)	0	1	2	3	4	5	6	7	8	9	10
0	-	-	-	-	-	-	-	-	-	-	-
1	<0.001	-	-	-	-	-	-	-	-	-	-
2	<0.001	<0.001	-	-	-	-	-	-	-	-	-
3	<0.001	<0.001	0.495	-	-	-	-	-	-	-	-
4	<0.001	<0.001	0.005	0.001	-	-	-	-	-	-	-
5	<0.001	<0.001	0.006	0.001	0.920	-	-	-	-	-	-
6	<0.001	<0.001	0.017	0.002	0.645	0.719	-	-	-	-	-
7	<0.001	<0.001	0.011	0.001	0.780	0.859	0.856	-	-	-	-
8	<0.001	<0.001	0.005	0.001	0.990	0.929	0.654	0.789	-	-	-
9	<0.001	<0.001	0.007	0.001	0.884	0.964	0.753	0.894	0.893	-	-
10	<0.001	<0.001	0.020	0.003	0.606	0.678	0.956	0.812	0.614	0.711	-
